# Sheep Manure-Tail Vegetable-Corn Straw Co-Composting Improved the Yield and Quality of Mini Chinese Cabbage

**DOI:** 10.3390/foods14020163

**Published:** 2025-01-08

**Authors:** Xuehua Wang, Yali Qiao, Jianzhong Tie, Wenbin Zhang, Baihong Wei, Zeci Liu, Jihua Yu, Linli Hu

**Affiliations:** 1 College of Horticulture, Gansu Agricultural University, Lanzhou 730070, Chinayujihua@gsau.edu.cn (J.Y.); 2 Gansu Provincial Key Laboratory of Arid land Crop Science, Gansu Agricultural University, Lanzhou 730070, China

**Keywords:** planting and breeding waste, mini Chinese cabbage, yield, quality

## Abstract

In order to fully utilize the resources of agricultural waste in Gansu Province’s semi-arid area. Local commercial organic fertilizer (ST1) was selected as the control, and four kinds of planting and breeding waste composts (PBCs) were designed with sheep manure (SM), cow manure (CM), tail vegetable (TV), mushroom residue (MR), and corn straw (CS) to study the effects of the different PBC formulations on the yield and quality of mini Chinese cabbage. In contrast to local commercial organic fertilizer, the STS (SM:TV:CS = 6:3:1) treatment increased the economic yield by 5.56%. Additionally, STS also significantly increased the VC content of mini Chinese cabbage, increased the organic acid by 14.66%, increased the free amino acid by 38.98%, and the nitrate concentration was significantly reduced by 41.05%. Meanwhile, the STS formula also increased the concentrations of polyphenols and essential amino acids of mini Chinese cabbage and also had excellent performance in volatile compounds. As a result, the STS formula can make full use of local planting and breeding waste resources and produce high yield and high quality of local mini Chinese cabbage. The study provided a theoretical foundation and technical guidance for screening suitable local compost formulas, as well as for the achievement of high-yield and high-quality mini Chinese cabbage production in the semi-arid areas of central Gansu province.

## 1. Introduction

Mini Chinese cabbage (*Brassica rapa* ssp. *Pekinensis*), which belongs to the *Brassica* family and prefers cold and clammy in nature, is a key vegetable variety cultivated during summer in the Chinese plateau. In recent years, the planting area in the north of China has also gradually increased. As individuals seek a higher quality of life, their expectations for both the quantity and quality of vegetables have increased. Consequently, it is urgent to enhance the yield and quality of *Brassica rapa* ssp. *Pekinensis* [1]. One way of ensuring a high crop yield is by fertilization, and effective fertilization can guarantee both the quality of agricultural goods and healthy crop growth. Additionally, the type of fertilizer utilized will affect crop growth and development. At present, in order to increase the productivity of mini Chinese cabbage, a great quantity of phosphorus, potassium, and nitrogen fertilizers are frequently applied. Excessive utilization of chemical fertilizers not only increased the nitrate content of vegetables, resulting in quality degradation, but also endangered human health. The application of excessive fertilizers has also caused risk impacts on the soil, such as soil crusting, imbalance of nutrient structure, harmful metals and pathogens exceeding the standard, and different types of environmental pollution [2]. Although chemical fertilizer has a pivotal role in increasing agricultural production and income, the application of chemical fertilizers has increased year by year, resulting in a decline in chemical fertilizer use efficiency, and environmental problems have become more and more prominent [3]. People have blindly used fertilizers to increase production, which significantly pollutes the environment in addition to increasing the expense of agricultural production. posing a challenge to the quality and safety of agricultural products [4]. To reduce the negative impacts of excessively chemical fertilizer application, there is a need to reduce amounts of chemical fertilizers used to improve the quality and yield of *Brassica rapa* ssp. *Pekinensis* [5]. Using organic fertilizers in place of chemical fertilizers not only helps to increase soil fertility and increase crop yield but also restores the richness of microbial flora in the soil [6], and compost is a kind of organic fertilizer [7].

China is a large agricultural production country. The rapid increase in agricultural output value, in the meantime, has produced a large amount of agricultural waste, mainly from planting and breeding. Planting and breeding waste composts (PBCs) refers to the organic material discarded in the process of planting and breeding production, mainly including crop residues from planting production and livestock and poultry manure from farming production, such as plant or animal residual waste [8]. Therefore, accelerating the resource utilization of planting and breeding waste (PBW) is the key, and composting treatment is an effective way to utilize it [9]. Which is a controlled biological process that uses microorganisms in aerobic conditions to transform organic waste into organic manure [10], recycling mineral nutrients (nitrogen (N), phosphorus (P), and potassium (K)) that could be used in agriculture [11]. The composting process of PBW can produce biochar. A study found that biochar, which is alkaline, highly porous, and contains mineral elements such as N, P, K, Ca (calcium), Mg (magnesium), etc., which are necessary for plant growth, when used as a soil conditioner, can reduce soil acidity, increase soil fertility, and improve the soil granular structure and the environment for microbial growth and reproduction as well as for the growth of crop roots [12]. In addition, different formula raw materials could have an impact on compost and plants. Tomatoes treated with *Trichoderma*-enriched bio-organic fertilizer accumulated up to 24%, 57%, and 62% of total soluble sugars, vitamin C, and nitrate, respectively, compared to conventional chemical fertilizers. A research study found that a cassava peel and corncob matrix mixture (1:1, V:V) with added chicken manure compost could improve the yield of *Pleurotus ostreatus* [13]. Kang et al. [14] showed that different concentrations of kitchen waste mixed with food waste fertilizer treatment favored the growth of Chinese cabbage, as evidenced by a significant increase in chlorophyll content and mineral content. Meanwhile, the application of PBCs could not only inhibit pathogenic bacteria but also improve the structure of microbial communities, which can form the “biological wall” around the root system in the continuous crop, as well as promote a variety of enzyme activity of the soil [15,16]. As a result, PBCs could solve the problem of agricultural waste accumulation. Furthermore, it will have different effects on crops due to different raw materials.

## 2. Materials and Methods

### 2.1. Experiment Site

The experimental site was situated at the plateau summer vegetable planting base in the Gansu Kangyuan Modern Agriculture Co., Ltd. in Yuzhong County, Lanzhou City (35°87′ N, 104°23′ E), characterized by a temperate semi-arid continental climate. The average altitude is about 1790 m, with a yearly average temperature of 6.6 °C, and the frost-free period lasts approximately 150 d. Annual precipitation ranges from 300 to 400 mm, and the evaporation is about 1343 mm. The experimental field features mild topography, yellow spongy soil, and uniform fertility. The soil’s physical and chemical characteristics were shown in Table 1.

### 2.2. Study Material

Test varieties: mini Chinese cabbage (*Brassica rapa* ssp. *Pekinensis*, var. ‘Empire’)

Raw materials for composting fermentation: sheep manure, tail vegetable, cow manure, mushroom residue, corn straw, and the nutrients of the raw materials were shown in Table 2.

### 2.3. Experimental Design

#### 2.3.1. Compost Formula Design

ST1 (SM:TV = 6.5:3.5)

ST2 (SM:TV = 5.5:4.5).

STC (SM:TV:CM = 6:3:1).

STM (SM:TV:MR = 6:3:1).

STS (SM:TV:CS = 6:3:1).

The basic physicochemical properties of different planting and breeding waste compost were determined as follows Table 3.

#### 2.3.2. Compost Fermentation

The basic physical and chemical properties of different PBCs were shown in Table 3. Following the design formula, the raw ingredients were blended, and the pile’s water content was adjusted to between 60 and 65 percent. With a height of one meter, a bottom width of two meters, a top width of one meter, and an infinite length, stack them into a table-shaped pile (the cut surface is trapezoidal). Monitor the temperature at a distance of 25 cm from the top of the pile. Turn the pile for the first time when the temperature is above 60 °C, and then once every seven days after that. The pile will turn brown, have a faint grassy odor, and have reached about room temperature after 40 days of fermentation.

### 2.4. Experiment Method

All treatments were applied at one time before planting. Each treatment had 3 plots with an area of 16 m^2^ (16 m × 1 m). The test was carried out on 3 July 2021 with 72-hole plug trays, and on July 25, select the seedlings with the same size, no pests and diseases, and grow vigorously for planting, with 106 plants in each plot. On the planting day, seedlings were watered once, and the cultivation mode of one ridge and two rows of film mulching was adopted. The ridge width was 1 m, the ridge length was 16 m, and the furrow width was 40 cm. Protective rows were set up. Triangular planting with a plant spacing of 30–35 cm and plant row spacing of 20–25 cm, a total of 2946 mini Chinese cabbages were planted per acre. Field management measures such as irrigation and pest control throughout the growth period are consistent with local traditional management measures.

### 2.5. Yield Determination

After the product organs reached maturity (65 days after transplanting), the equidistant method was used for random sampling after removing the marginal effect. The biological yield (the weight of the whole mini Chinese cabbages) and economic yield (the cabbage retained after removing the roots and the large green outer leaves from the outside) were determined by simple random sampling of 10 mini Chinese cabbages from each plot.

### 2.6. Quality Determination

At harvest time, 10 mini Chinese cabbages were randomly selected from each plot; after removing the non-edible part, the edible part was separated into tetrads. Then free amino acids, soluble sugar, organic acid, soluble protein, nitrate, and vitamin C (VC) contents were determined following fruit grinder homogenization and grinding. The content of free amino acid was determined from 1.00 g of leaf using the Ninhydrin reaction. Fresh frozen sample was heated in a water bath for 30 min at 80 °C with 10 mL deionized water. After centrifuging at 13,000× *g* for 10 min. The 0.2 mL supernatant was mixed with 19 mL NaOH (4 mol·L^−1^) and 0.8 mL salicylic acid (5% (*w*/*v*)). The absorbance of the mixture was determined at 410 nm by a UV spectrophotometer. Soluble sugar content was determined from 0.50 g of leaf using the anthrone colorimetric method; fresh frozen samples were heated for 30 min in a boiling water bath with 10 mL of distilled water. Then, 0.1 mL supernatant was mixed with 1.9 mL distilled water, 5 mL vitriol, and 0.5 mL anthrone ethyl acetate. After shaking and cooling to ambient temperature, the solution was measured by the UV-spectrophotometer at 630 nm [17]. The organic acid was determined from 5.00 g of leaf using acid-base titration. Fresh frozen tissue was ground to a fine paste and placed in an Erlenmeyer flask. Deionized water was then added to a volume of 50 mL, and the mixture was filtered. The filtrate then underwent titration with 0.1 mol⋅L^−1^ sodium hydroxide (containing two drops of 1% phenolphthalein), resulting in a faint pink color, which was used as an indicator. Mean values were calculated from three independent sample replicates [18]. Soluble protein content was measured from 2.00 g of leaf by the Coomassie brilliant blue G-250 method. Fresh frozen tissue was ground up by a mortar and pestle with liquid nitrogen and then added into 5 mL distilled water. After centrifuging at 10,000 rpm for 10 min, 0.5 mL of supernatant was diluted in the same volume of distilled water and added into 4 mL of Coomassie brilliant blue G-250 solution, then examined by a UV-spectrophotometer at 595 nm [19]. Using the 2,6-dichloroindophenol stain method, Vitamin C content was measured from 0.50 g of leaf. Fresh frozen tissue was treated with 2 mL of 1% metaphosphoric acid and filtered. Then, the filtrates were mixed with 9 mL of 2, 6-dichloroindophenol (0.15 mg mL^−1^), and absorbance was measured at 515 nm. Ascorbic acid content of the plant sample was calculated on the basis of the calibration curve and was expressed in mg 100 g^−1^. The concentration range of ascorbic acid used for the standard curve was 0.3–3.5 µg mL^−1^ [20]. Using the salicylic acid-sulfuric acid method, the nitrate content was measured from 3.00 g of leaf. Fresh frozen tissue was weighed in a test tube, 10 mL of ultrapure water was added, sealed, and extracted in boiling water for 30 min, then cooled, filtered, and rinsed the residue repeatedly, and the volume was set to 25 mL. 0.1 mL of the supernatant was sucked up and added to 0.4 mL of 5% salicylic acid-sulfuric acid solution, mixed, and then left to stand at room temperature for 20 min, and then slowly added to 9.5 mL of 8% NaOH solution and mixed well. After cooling, the absorbance value at 410 nm was measured [21].

Amino acid fractions and polyphenols were referred to liquid chromatography-mass spectrometry (LC-MS) with 0.1 g of mini Chinese cabbage freeze-dried samples according to Jin et al. [22]. Quantification using HPLC-MS (LC-MS, Agilent 1290-6460, Santa Clara, CA, USA), chromatographic column: Agilent InfinityLab Poroshell 120 HILIC-Z column (2.1 × 100 mm, 2.7 μm); The column temperature was 25 °C and the flow rate was 0.5 mL·min^−1^. The mass spectrometry source conditions are as follows: the ionization mode is the ESI positive ion mode; the dry gas temperature is 330 °C; the gas flow rate is 13 L·min^−1^; atomizer is 35 psi; the sheath gas temperature is 390 °C; the sheath gas velocity is 12 L·min^−1^; and the capillary voltage is 1500 v. The 18 free amino acids (FAAs) contained 9 essential amino acids (EAAs) and 9 non-essential amino acids (NEAAs) [23]. Among them, EAAs are Threonine, Isoleucine, Tryptophan, Valine, Phenylalanine, Leucine, Methionine, Lysine, and Arginine. Nine NEAAs are Tyrosine, Glycine, Glutamate, Asparagine, Proline, Cysteine, Alanine, Serine, and Glutamine. The medicinal amino acids (MAAs) [24] include Phenylalanine, Leucine, Methionine, Arginine, Lysine, Tyrosine, Glycine, Glutamate, Asparagine, and nine others.

Polyphenols were determined by referring to the method of Jin et al. [22]: A total of 0.1 g of cabbage freeze-dried powder and 2 mL of methanol were added into a 5 mL centrifuge tube and placed at room temperature for 1 h for extraction. The samples were centrifuged at 4 °C and 8000 rpm for 10 min, and then the supernatant was filtered with a 0.22 µm organic phase filter membrane. A total of 10 μL of the sample were aspirated, and a symmetrical C18 column (250 mm × 4.6 mm, 5 μm, Waters Corp., Milford, MA, USA) was used for HPLC analysis. The flow rate was 1.1 mL·min^−1^, the mobile phase was methanol (A) and 1% (*v*/*v*) acetic acid (B), and the column temperature was maintained at 30 °C. Gradient elution was used.

Volatile compounds were determined by using the electronic nose analyzer (PEN3, Airsense Analytics GmbH, Schwerin, Germany). The types and performances of used electronic nose sensors were shown in Table 4. Refer to Wang’s [25] method, slightly changed: A quantity of 0.5 g homogenized fresh mini-Chinese cabbage samples and 1.5 g anhydrous sodium sulfate were added to the headspace vials, which were then tightly capped and placed on a magnetic mixer at 70 °C for 15 min to achieve internal headspace gas equilibrium. Subsequently, an injection needle was then inserted into the headspace vial for the purpose of quantifying the volatile compounds. The conditions for the e-nose test were as follows: the rinse time was 60 s, the sensor zero time was 5 s, the pre-sample time was 5 s, the injection flow was 400 mL·min^−1^, and the measurement time was 120 s. One mini Chinese cabbage was measured in each plot of the five treatments, repeated three times, and a total of 3 mini Chinese cabbages were determined, 5 × 3 × 3 times, and the mean value of each cabbage was used for statistical analysis.

### 2.7. Statistical Analysis

The yield per hectare of each plot was calculated from the obtained individual plant weight and planting density, then the average of each quadrat was calculated, and these aggregated data were used for statistical analysis. The quality determinations were calculated according to the absorbance of three independent biological samples. Principal component analysis (PCA) and correlation analysis used compost formula as independent variables and amino acid components and phenolic acid types as dependent variables. The mean values of three independent biological experiments were used to plot the data to study which of the amino acid and phenolic acid components in this experiment is the main component and what is the correlation between each component. Microsoft Excel 2019 software was used to process the data, SPSS 25.0 software was used to perform one-way ANOVA with principal component analysis, Duncan’s test was used for multiple comparisons of significant differences (*p* < 0.05), and Origin 2024 software was used for graphing.

## 3. Results

### 3.1. Effects of Different PBCs on Yield in Mini Chinese Cabbage

**Figure 1:**
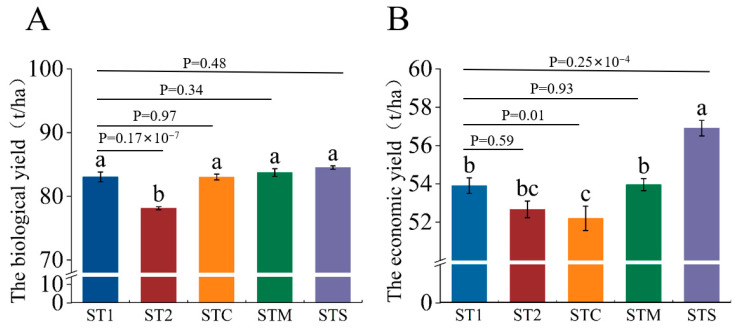
Effects of different formulations of PBCs on biological yield (**A**) and economic yield (**B**) in mini Chinese cabbage. Abbreviations: ST1 (SM:TV = 6.5:3.5). ST2 (SM:TV = 5.5:4.5). STC (SM:TV:CM = 6:3:1). STM (SM:TV:MR = 6:3:1). STS (SM:TV:CS = 6:3:1). Data were means ± SE (n = 10) from three independent replications. Different lowercase letters above the error bars presented significant differences among treatments at the 0.05 level (*p* < 0.05) according to Duncan’s multiplied test; the size of the effect is 0.685 (**A**) and 0.594 (**B**).

The biological yield of mini Chinese cabbage was not significantly different among ST1, STC, STM, and STS, but those were significantly higher than that in the ST2 treatment (Figure 1A). The economic yield of cabbage treated with ST1 was significantly higher than that treated with STC, while not significantly different from those treated with ST2 and STM but significantly lower than that treated with STS (Figure 1B). Moreover, both the biological and economic yields of cabbage treated with STS reached the highest values, which were increased by % and 5.56% compared to ST1, respectively (Figure 1).

### 3.2. Effects of Different PBCs on the Quality in Mini Chinese Cabbage

**Figure 2:**
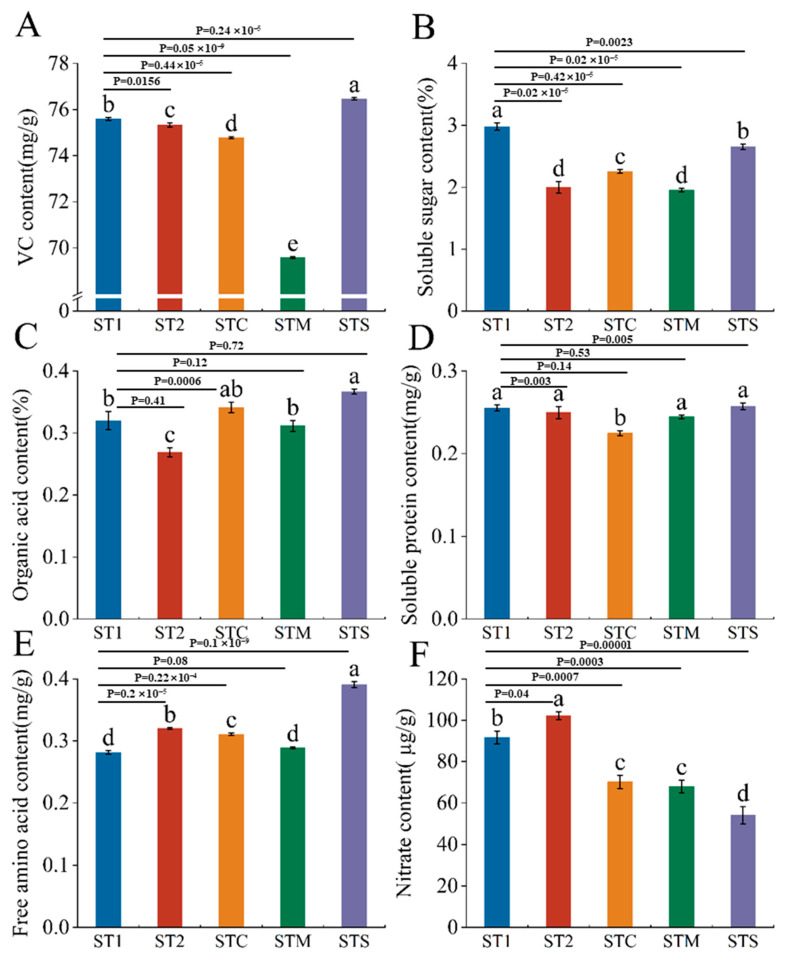
Effects of different formulations of PBCs on the contents of VC (**A**), soluble sugar (**B**), organic acid (**C**), soluble protein (**D**), free amino acid (**E**), and nitrate (**F**) in mini Chinese cabbage. Abbreviations: ST1 (SM:TV = 6.5:3.5). ST2 (SM:TV = 5.5:4.5). STC (SM:TV:CM = 6:3:1). STM (SM:TV:MR = 6:3:1). STS (SM:TV:CS = 6:3:1). Data were means ± SE (n = 3) from three independent replications. Different lowercase letters above the error bars presented significant differences among treatments at the 0.05 level (*p* < 0.05) according to Duncan’s multiplied test. The size of the effect is 0.999 (**A**), 0.960 (**B**), 0.778 (**C**), 0.859 (**D**), 0.990 (**E**), and 0.938 (**F**).

As shown in Figure 2, STS significantly increased the VC content by 1.15% of mini Chinese cabbage compared to ST1, while the rest of the treatments reduced the VC content of mini Chinese cabbage, especially the STM treatment (Figure 2A). The content of soluble sugar was significantly reduced in all treatments compared to ST1, where that under STS formulas was significantly higher than the other formulas (Figure 2B). Compared with ST1, STS and STC increased organic acids by 14.66% and 6.66%, respectively (Figure 2C). Only STC showed a substantial decrease in the amount of soluble protein; the other treatments did not differ significantly (Figure 2D). STS outperformed the other treatments, dramatically reducing nitrate concentration by 41.05% (Figure 2F) and increasing free amino acids by 38.98% as compared to ST1 (Figure 2E). Finally, compared to ST1 treatment, STS significantly increased the contents of VC, organic acid, and free amino acid, as well as significantly decreased the nitrate accumulation in *Brassica rapa* ssp. *Pekinensis*.

### 3.3. Effects of Different PBCs on the Amino Acid Components in Mini Chinese Cabbage

#### 3.3.1. Variance Analysis

The total free amino acids (TFAAs) of each treatment ranged from 142.04 to 187.59 g/kg (Table 5). Under ST2 treatment, TFAAs, NEAAs, and MEAAs were significantly higher than those of other treatments, and the amino acid content, except for Glutamine, was significantly higher than that of ST1 treatment. The contents of Tryptophan, Valine, Arginine, Glycine, Asparagine, Proline, Cysteine, and Alanine were higher than those under the other formulations. Methionine and Glutamine under the STC were the largest. Tryptophan, Glutamate, Asparagine, and Cysteine in STM formulation were higher than those in ST1, and Glutamate content was the maximum among treatments. The content of EAAs in all treatments ranged from 53.37–76.56 g/kg, and the highest content of essential amino acids was found in mini Chinese cabbage under STS treatment, which was significantly increased by 18.28% compared with ST1. Under STS treatment, the contents of the amino acids except for Phenylalanine, Glutamate, and Proline were significantly higher than those of ST1. Also, Threonine, Isoleucine, Phenylalanine, Leucine, Lysine, Tyrosine, and Serine contents were maximum in all treatments. The contents of NEAAs ranged from 75.86–95.19 g/kg. Under ST2 treatment, the content of NEAAs was the highest, which was 25.48% higher than ST1. In addition, the content of MAAs in mini Chinese cabbage under ST2 treatment ranged from 104.46–130.62 g/kg and was highest in ST2 treatment, with a significant increase of 18.26% compared with ST1.

#### 3.3.2. Principal Component Analyses (PCA)

**Figure 3:**
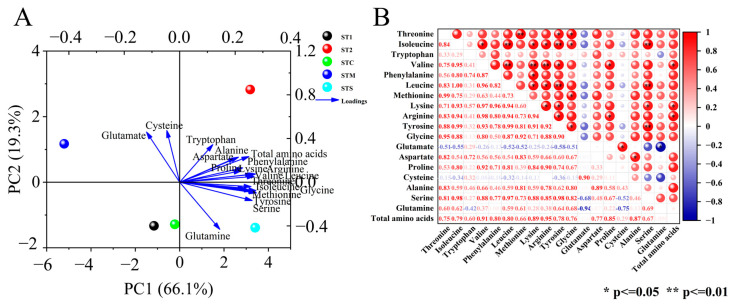
Principal component analysis (**A**) and correlation analysis (**B**) of amino acids in mini Chinese cabbage under different PBCs. The data were expressed as average values (n = 3). The * and ** represent significant correlations at *p* < 0.05 and *p* < 0.01 levels, respectively (two-tailed). The red indicated positive correlation, and blue indicated negative correlations. Abbreviations: ST1 (SM:TV = 6.5:3.5). ST2 (SM:TV = 5.5:4.5). STC (SM:TV:CM = 6:3:1). STM (SM:TV:MR = 6:3:1). STS (SM:TV:CS = 6:3:1).

The model of the influence of different PBCs on the amino acid components of mini Chinese cabbage based on PCA was shown in Figure 3A, with five treatments and 18 amino acid components forming the corresponding groups. The first two principal components had a variance contribution rate of 85.4%, of which PC1 and PC2 explained 66.1% and 19.3% of the total variance, respectively. In addition, the loading diagram showed that Arginine has the strongest first principal component with Isoleucine, followed by Methionine and Valine, and Glutamate has the strongest second principal component with Cysteine. Therefore, it can be used as a representative factor to reflect the composition and content of amino acids in mini Chinese cabbage under different PBCs. Meanwhile, each treatment had obvious secession based on both PC1 and PC2; the fractions and contents of amino acids in mini Chinese cabbage under ST1 and STC treatments, which were placed in the same quadrant, were also more similar.

#### 3.3.3. Correlation Analysis

The results of correlation analysis (Figure 3B) showed multiple sets of highly significant correlations between the amino acid constituents of mini Chinese cabbage under different PBCs. Among them, highly significant positive correlations were discovered between Threonine and Methionine (r = 0.99), as well as between Isoleucine and Leucine (r = 1.00), Tyrosine (r = 0.99), and Serine (r = 0.98). Valine was found to be highly significantly positively correlated with Leucine (r = 0.96), Lysine (r = 0.97), and Arginine (r = 0.98). Leucine had significantly positive correlations with both Serine (r = 0.97) and Tyrosine (r = 0.99). Tyrosine was highly significantly positively correlated with Serine (r = 0.98). There were also several sets of significant positive correlations, including those involving Threonine and Tyrosine (r = 0.88), Glycine (r = 0.95); Isoleucine and Valine (r = 0.95), Lysine (r = 0.93), Arginine (r = 0.94), Glycine (r = 0.88) were significantly positively correlated with each other; Valine was significantly positively correlated with Tyrosine (r = 0.93), Proline (r = 0.92), Total amino acids (r = 0.91); Methionine and Glycine (r = 0.92) also showed a significant positive correlation, the Arginine was significantly positively correlated with Tyrosine (r = 0.92), Proline (r = 0.90) and Total amino acids (r = 0.95); Glutamate was significantly positively correlated with Cysteine (r = 0.90) and significantly negatively correlated with Glutamine (r = −0.94).

### 3.4. Effects of Different PBCs on Polyphenols in Mini Chinese Cabbage

#### 3.4.1. Variance Analysis

Fifteen kinds of polyphenols (Table 6) were identified in mini Chinese cabbage, including 11 kinds of phenolic acids (protocatechuic acid, p-hydroxybenzoic acid, chlorogenic acid, gallic acid, coumalic acid, ferulic acid, benzoic acid, cinnamic acid, caffeic acid, sinapic acid, gentisic acid) and four flavonoids (rutin, quercetin, cynarin, kaempferol). With the exception of gallic acid, cinnamic acid, sinapic acid, and kaempferol, the contents of polyphenol under ST2 treatment were lower than those under ST1. The contents of phenolic acids and flavonoids in mini Chinese cabbage under ST1 formulas were significantly higher than those under STC and STM formulas. Nevertheless, under STS formulas, except for protocatechin acid and rutin, other phenolic acids were the highest in all treatments and significantly higher than the ST1 treatment. In addition, the contents of total phenolic acids and total flavonoids were also the highest under STS treatment. Under STS treatment, the highest content of phenolic acids was sinapic acid, which reached 286.71 ug/g DW, accounting for 19.89% of the total phenolic acids. The highest content of flavonoids was quercetin, which reached 1154.99 ug/g DW, accounting for 69.74% of the total flavonoids.

#### 3.4.2. Principal Component Analyses

**Figure 4:**
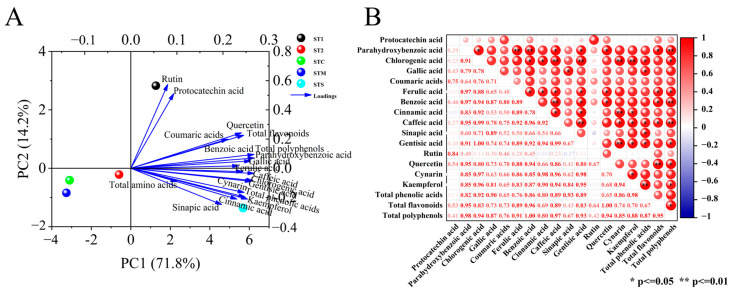
Principal component analysis (**A**) and correlation analysis (**B**) of polyphenols in mini Chinese cabbage under different PBCs. The data were expressed as average values (n = 3). The * and ** represented significant correlations at *p* < 0.05 and *p* < 0.01 levels, respectively (two-tailed). The red indicated positive correlation, and blue indicated negative correlation. Abbreviations: ST1 (SM:TV = 6.5:3.5). ST2 (SM:TV = 5.5:4.5). STC (SM:TV:CM = 6:3:1). STM (SM:TV:MR = 6:3:1). STS (SM:TV:CS = 6:3:1).

The PCA of polyphenols in mini Chinese cabbage under different PBCs are shown in Figure 4A. The variance contribution rate of the first two principal components reached 89.9%, of which PC1 and PC2 explained 74.6% and 15.3% of the total variance, respectively. In addition, it can be seen from the load diagram that caffeic acid has the strongest first principal component complex, and rutin and protocatechin acid have the strongest second principal component complex. Therefore, it can be used as a representative factor to reflect the polyphenols of mini Chinese cabbage under different PBCs. Meanwhile, ST1 produced an obvious separation from other treatments based on PC1, and ST1 and STS produced an obvious separation from other treatments based on PC2, with STC being more similar to the STM treatment.

#### 3.4.3. Correlation Analysis

As shown in Figure 4B, multiple sets of highly significant and significant positive correlations between polyphenols were revealed by Pearson’s correlation analysis. Among them, there was a significant positive correlation between parahydroxybenzoic acid and ferulic acid (r = 0.97) and benzoic acid (r = 0.97); and chlorogenic acid was significantly positively correlated with caffeic acid (r = 0.99), gentisic acid (r = 1.00), cynarin (r = 0.97), and kaempferol (r = 0.96). Caffeic acid and gentisic acid had a significant positive correlation (r = 0.99). Parahydroxybenzoic acid and chlorogenic acid (r = 0.91), caffeic acid (r = 0.95), gentisic acid (r = 0.91), and quercetin (r = 0.95) showed a significant positive correlation. chlorogenic acid was positively correlated with benzoic acid (r = 0.94) and cinnamic acid (r = 0.92). Ferulic acid was positively correlated with benzoic acid (r = 0.89), cinnamic acid (r = 0.89), caffeic acid (r = 0.92), gentisic acid (r = 0.89), and quercetin (r = 0.88). There was a positive correlation between cinnamic acid and kaempferol (r = 0.90), gentisic acid (r = 0.94), and caffeic acid (r = 0.92). Caffeic acid was significantly positively correlated with cynarin (r = 0.96) and kaempferol (r = 0.94). There was no significant negative correlation between polyphenols.

### 3.5. Effects of PBCs on the Volatile Compounds of Mini Chinese Cabbage

#### 3.5.1. Variance Analysis

**Figure 5:**
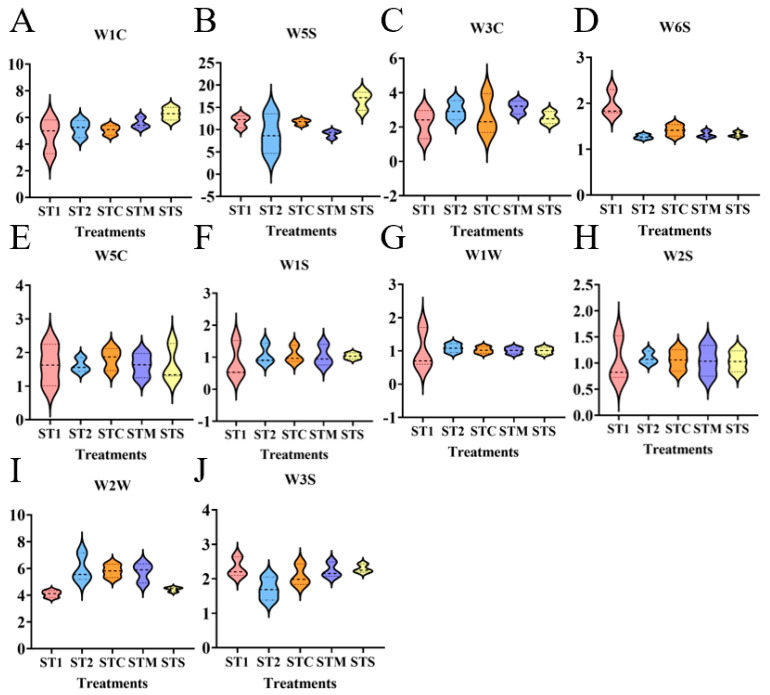
Violin plot of volatile compounds in mini Chinese cabbage under different PBCs. The response value of mini Chinese cabbage to W1C-sensitive to aromatic compounds (**A**), W5S-high sensitivity, sensitive to nitrogen oxides (**B**), W3C-sensitive to ammonia and aromatic compounds (**C**), W6S-sensitive to hydrogen (**D**), W5C-sensitive to olefins and aromatic compounds (**E**), W1S-sensitive to hydrocarbons (**F**), W1W-sensitive to hydrogen sulfide (**G**), W2S-sensitive to alcohols and some aromatic compounds (**H**), W2W-sensitive to aromatic compounds and organic sulfur compounds (**I**) and W3S-sensitive to alkanes (**J**) under different PBC. Abbreviations: ST1 (SM:TV = 6.5:3.5). ST2 (SM:TV = 5.5:4.5). STC (SM:TV:CM = 6:3:1). STM (SM:TV:MR = 6:3:1). STS (SM:TV:CS = 6:3:1).

The electronic nose sensor was used to analyze the volatile compounds of mini Chinese cabbage under the different PBC treatments. As shown in Figure 5, all 10 types of electronic nose sensors responded to the volatile compounds of mini Chinese cabbage, especially the three types of nitrogen oxides (W5S) (Figure 5B), aromatic compounds (W1C) (Figure 5A), and aromatic compounds and organic sulfides (W2W) (Figure 5I), which were more sensitive to the response of the electronic nose sensors, while the remaining seven substances had small response values for the electronic nose sensor, indicating that the flavor of mini Chinese cabbage is mainly determined by W5S, W1C, and W2W. Among them, W5S accounted for the largest proportion of the volatile compounds of mini Chinese cabbage, and the response values of both W5S and W1C were higher than those of the other treatments under the STS treatment.

#### 3.5.2. Principal Component Analyses

**Figure 6:**
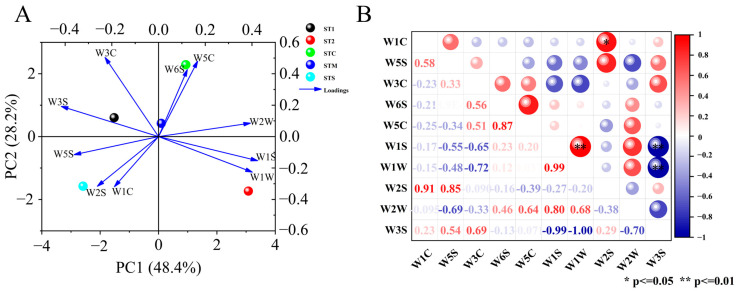
Principal component analysis (**A**) and correlation analysis (**B**) of response value of volatile compounds in mini Chinese cabbage under different PBCs. The data were expressed as average values (n = 3). The * and ** represented significant correlations at *p* < 0.05 and *p* < 0.01 levels, respectively (two-tailed). The red indicated positive correlation, and blue indicated negative correlation. Abbreviations: ST1 (SM:TV = 6.5:3.5). ST2 (SM:TV = 5.5:4.5). STC (SM:TV:CM = 6:3:1). STM (SM:TV:MR = 6:3:1). STS (SM:TV:CS = 6:3:1).

PCA of flavor substances was performed to assess the effect of different compost treatments on the grouping of mini Chinese cabbage. The scores and loadings were shown in Figure 6A, with the first two principal components contributing 76.6%, with PC1 and PC2 explaining 48.4% and 28.2% of the total variance, respectively. In addition, the loading diagram showed that W1S had the strongest first principal component, and W3C had the strongest second principal component. Therefore, it could be used as a representative factor to reflect the flavor substances of mini Chinese cabbage under different PBCs. At the same time, each treatment based on PC1 and PC2 produced a significant separation.

#### 3.5.3. Correlation Analysis

Pearson’s correlation analysis of flavor substances (Figure 6B) showed significant positive correlation between W1C and W2S (r = 0.91) and highly significant positive correlation between W1S and W1W (r = 0.99). It was discovered that there was a highly significant negative correlation between W1S and W3S (r = −0.99), as well as an extremely significant negative correlation between W1W and W3S (r = −1.00).

## 4. Discussion

### 4.1. Effect of Different PBCs on the Yield of Mini Chinese Cabbage

Composting is not only an effective measure to deal with agricultural waste but also effective in increasing crop yield and improving soil environment [26]. Due to the different compost raw materials and their different chemical components, their effects on soil physical and chemical properties, crop yield, and quality were also different when they were applied to soil [27]. The same results were found in our experiment; the biological and economic yields of mini Chinese cabbage were significantly decreased after applying compost that raised the portion of tail vegetables in comparison to the local commercial organic fertilizer. The study of Lu et. al. [28] showed that a single tail vegetable compost is not conducive to the growth of lignocellulose-degrading bacteria (*Aspergillus* and *Thermomyces*), which are the key colonies of promoting compost maturity. In addition, the main component of the tail vegetable is water; there is only a small amount of dry biomass, which can be mineralized to produce bioavailable nutrients [29]. In summary, the compost formula that improved the portation of tail vegetables was not only not conducive to compost maturity but also did not contribute to improving the available nutrient content of plants in composting. As a result, the ST2 formula reduced the yield compared with the local commercial organic fertilizer. The compost added with cow manure significantly reduced the economic yield of mini Chinese cabbage. This may be due to the fact that the cow feed (mainly grass) was treated by microorganisms in the rumen of the cow, so that CM had a higher lignocellulose content (cellulose, hemicellulose, and lignin), resulting in slower hydrolysis of CM during composting and faster nutrient loss [30]. Moreover, since there is a high concentration of animal manure in STC, there will be less pathogen inactivation during composting, which will indirectly impact plant growth and lower yield. [31]. The compost added with straw significantly increased the economic yield of mini Chinese cabbage. The possible reasons might be, on one hand, the abundance of microbial flora in STS was higher than that in ST1 after adding straw, which is helpful for plant growth. On the other hand, the addition of expansive fillers such as straw in compost was not only a good carbon source but also made the air more circulated and thus made the composting effect better [32].

### 4.2. Effect of Different PBCs on the Nutritional Quality in Mini Chinese Cabbage

The quantity and distribution ratio of soluble protein, soluble sugar, nitrate, organic acid, free amino acid, and Vc were used to evaluate the nutritional quality of mini Chinese cabbage [33]. Soluble sugar, one of the key evaluation indexes for mini Chinese cabbage, serves as a signal transduction molecule that controls development and allows plants to adapt to changing environmental conditions in addition to being a vital component for energy production and the bare carbon skeleton for a variety of metabolic pathways [34]. In addition, soluble sugars, soluble proteins, and free amino acids are also important osmotic regulators in plants. They play a significant role in plant stress response by regulating the osmotic pressure of cytoplasm to protect enzymes, proteins, and cell membranes. The nitrate was the precursor synthesis of nitrosamines, and excessive intake of nitrate would endanger human health and lead to digestive system cancer [17]. Organic acid is an indicator to evaluate the maturity and quality of vegetables and fruits [35]. Ascorbic acid content (Vitamin C) is one of the major quality components in mini Chinese cabbage [36] and is thought to reduce the risk of atherosclerosis, cardiovascular disease, and certain cancers [37]. Jin et al. [22] discovered that the use of organic fertilizer led to a significant increase in the content of tomatoes’ soluble protein, soluble sugar, and vitamin C, effectively reducing the content of nitrate, so that the quality of agricultural products was greatly improved, and we also obtained consistent results. In our study, compared to ST1, the contents of VC, organic acids, and free amino acids in mini Chinese cabbage treated with STS significantly increased, and the accumulation of nitrate was significantly reduced. This may be because STS formulations obtain a higher carbon-to-nitrogen ratio (C/N ratio) due to the addition of straw, and the carbon-to-nitrogen ratio is an important indicator of compost maturity. The carbon-to-nitrogen ratio in crop straw is high, and the degradation rate is slow, and the composting method of mixing straw with vegetables can obtain a suitable carbon-to-nitrogen ratio and achieve rapid microbial decomposition [38]. A study has found that when the carbon-to-nitrogen ratio reaches 30:1, it will promote the growth of functional microorganisms responsible for lignocellulose degradation (*Luteimonas*, *Sphingobium*, *Trichoderma*, *Chaetomium*, and *Rosellinia*) [39]. In this experiment, the STS formulation achieved a carbon to nitrogen ratio of 39:1, while none of the other formulations reached 30:1. Additionally, Niu et al. [40] found that the addition of three conditioners, wheat straw, coconut bran, and mushroom residue, to the tail vegetable could make the tail vegetable aerobic composting under low C/N conditions, and the temperature, pH, EC, and GI values meet the relevant organic fertilizer standards. The addition of wheat straw is more easily able to promote the production of humic acid and promote the formation of humus. Therefore, the sheep manure-tail vegetable-cron straw co-composting formula has more advantages than the sheep manure-tail vegetable compost in terms of carbon-to-nitrogen ratio. Secondly, nitrogen plays a crucial role in enhancing both the yield and quality of plants, but excessive accumulation of nitrate nitrogen will instead reduce plant yield and quality, while nitrate nitrogen content in plants is positively correlated with nitrate content [32]. Straw application can enhance microbial activity, which can adsorb and immobilize NH_4_^+^ in fertilizers and inhibit NH_4_^+^ nitrification, thus reducing the formation of nitrate nitrogen [41]. Therefore, compared with ST1, STS showed a decrease in nitrate content, while ST2 increased nitrate accumulation. Finally, the content of total nitrogen and total phosphorus in corn straw is higher. After composting, the soil nitrogen content, nitrogen fixation capacity, and carbon-to-nitrogen ratio can be increased, and the diversity and uniformity of the soil bacterial community will also be improved [42]. Our previous study [43] found that in zucchini production systems, chemical fertilizer reduction by 30% + 9000 kg ha^−1^ sheep manure-tail vegetable-cron straw co-composting treatment could increase the richness and metabolic capacity of bacterial communities and increase the abundance of *Proteobacteria*, a phylum with absolute dominance (up to 32.46%). Compared to other formulations, STS contained a large amount of organic matter, a variety of nutrients were more comprehensive, and the release of nutrients was more alleviated, which is not only conducive to nitrogen mineralization but also helps to reduce the content of nitrate as well as the synthesis of vitamin C, which is able to improve the plant’s resistance to adversity [44,45]. As a result, the STS formula can increase the C/N ratio of soil, regulate the transformation between ammonium nitrogen and nitrate nitrogen, increase the richness and activity of soil microorganisms, and then affect the yield and quality of *Brassica rapa* ssp. *Pekinensis*.

Amino acids, as one of the most important indicators of the nutritional composition of vegetables, are involved in many important physiological processes such as protein synthesis, maintenance of nitrogen balance, regulation of physiological functions, energy supply, and replenishment of metabolic consumption in vegetables [46]. The content and type of free amino acids are also often used as one of the important indicators for evaluating the nutritional value and flavor of foods [47]. In this study, the contents of TFAAs, NEAAs, and MEAAs under ST2 treatment were significantly higher than those under other treatments, and the content of EAAs under STS treatment was the highest. In addition, the content of lysine was the highest, which is involved in the synthesis of nucleoproteins and hemoglobin and promotes the regeneration of cerebral neuronal cells and has a certain auxiliary therapeutic effect on the human body for malnutrition, hepatitis B, bronchitis, etc. [48]. The proportion of EAA content to total amino acid content in *Brassica rapa* ssp. *pekinensis* was around 40%, which is similar to the results of previous studies on a variety of native vegetables [46]. This may be because the lower pH of the ST2 formula and the higher organic matter content of STS affect the soil phosphatase activity and humus content, while phosphatase contributes to the formation of amino acid precursors, and humus has the ability to bind heavy metals and polar aromatic compounds, which can improve soil fertility [49]. These together affect the amino acid synthesis and change of mini Chinese cabbage. Sun found that the treatment of adding straw will make the corn root secrete more amino acids than the treatment without adding straw [50]. In addition, microorganisms and carbon sources are also important for the synthesis of amino acids [51]. Different compost raw materials lead to different microbial communities and carbon sources, so the number of amino acids is different between treatments. Arginine regulates the metabolism of energy, amino acids, and microbes through its involvement in the tricarboxylic acid cycle [52]. Isoleucine is the precursor of the bioactive molecule of jasmonates (JA-Ile), which is the active molecule of the plant hormone jasmonate (JA). In addition, JA is essential for multiple plant defense responses to biotic and abiotic stresses [53]. In our principal component analysis, they had the strongest first principal component loading and were correlated with some amino acids.

Eating vegetables rich in secondary metabolites, such as carotenoids, glucosinolates, anthocyanins, flavonoids, and polyphenols over the long term, has been associated with a reduced risk of developing various chronic diseases, including cancer and cardiovascular disease [54]. Phenolic substances play a key role in the appearance quality, nutritional and flavor quality of fruits and vegetables, antioxidants, anti-inflammatory, anti-allergic, effective prevention of high blood lipids, cardiovascular and cerebral vascular, hyperglycemia, etc., and have attracted widespread attention in many fields, including pharmaceuticals and food products [55]. Polyphenols contain phenolic acids, flavonoids, anthocyanins, tannins, etc. [56]. This experiment revealed the effect of different PBCs on the content of phenolic acids and flavonoids in mini Chinese cabbage, and a total of eleven phenolic acids and four flavonoids were detected in the mini Chinese cabbage, in which the highest content of phenolic acids was sinapic acid, which was in agreement with the study of Seong et al. [57], and sinapic acid is the characteristic substance in mini Chinese cabbage [58]. Quercetin, which accounts for the largest proportion of flavonoids, is an important factor in nutritional quality. The content of phenolic acids and flavonoids was the highest under the STS formula, indicating that STS treatment significantly increased the accumulation of polyphenolic substances in *Brassica rapa* ssp. *pekinensis*, which in turn improved the antioxidant capacity. However, they were relatively small under STC and STM treatments. Interestingly, the phenolic acid content of ST2 was higher than that of ST1, but the flavonoid content was significantly lower than that of ST1. The reason might be that the richness of soil microbial flora under the STS formula was relatively higher. Polyphenols are the products of lignin degradation and the precursors of the polyphenol humification pathway, and fungi and bacteria play an important role in lignin degradation [59]. A research study also found that adding plant growth-promoting compost (inoculated with Trichoderma harzianum, Bacillus subtilis, Pseudomonas aeruginosa, etc.) to compost could increase the content of total flavonoids and total polyphenols in plants [60]. We speculated that the polyphenol content in STC and STM was less since the lignin content in the two fomulas, adding CM and MS was less. Therefore, the polyphenol content was the highest under the STS formula. The study of Zhao et al. showed that pH during composting affected the continuous decomposition of lignocellulose by microorganisms [61]. In our experiment, after composting, the pH of ST2 was 7.95, while that of ST1 was 8.14. It is speculated that due to the high content of the tail vegetables in the ST2 formula, the pH of the compost changes greatly due to the incomplete decomposition of the tail vegetables in the compost fermentation process, which stimulates the key enzyme activity in the flavonoid synthesis pathway, resulting in a higher flavonoid content than the ST1 formula.

Volatile compounds are closely related to the flavor and nutrition of vegetables and can be used as indicators for evaluating the nutritional quality of mini Chinese cabbage. An electronic nose is a tool that simulates human olfactory perception. It can identify and classify volatile mixtures and is widely used to analyze food flavors. It is characterized by low cost and a high degree of automation [22,62]. In this experiment, 10 volatile compounds were detected by an electronic nose. Among them, nitrogen oxides (W5S), aromatic compounds (W1C), and aromatic compounds and organic sulfides (W2W) together determine the volatile compounds of *Brassica rapa* ssp. *pekinensis*. In addition, nitrogen oxides (W5S) accounted for the largest proportion in the flavor of mini Chinese cabbage as an important factor in nutritional quality. It is possible that the increase in the level of nitrogen accumulation in the PBC promotes the expression of flavor key genes and the increase in the content of volatile flavor substances, which significantly improves the flavor of mini Chinese cabbage [63,64].

In this experiment, different composting formulas can improve the yield and quality of mini Chinese cabbage in different degrees. Unfortunately, due to the northern climate, some wastes, such as fresh tail vegetables, cannot be supplied throughout the year. Therefore, we are also looking for other waste resources that can replace tail vegetables, such as kitchen waste, to set up more formulations. Finally, we hope to achieve a waste-compost-production cycle model through continuous resource utilization of waste.

## 5. Conclusions

1. In contrast to local commercial organic fertilizer, STS compost increased the economic yield by 5.56% of mini Chinese cabbage.
2. STS also significantly increased the VC content, EAA, and polyphenol contents of mini Chinese cabbage, increased the organic acid by 14.66%, increased the free amino acid by 38.98%, and the nitrate concentration was significantly reduced by 41.05%.
3. STS formula was the optimized compost for obtaining high yield and high-quality mini Chinese cabbage. It not only realizes the fertilizer utilization of the PBW in the Yuzhong area, reducing the environmental pollution, but also has the effect of improving the yield and quality of the local *Brassica rapa* ssp. *Pekinensis*. Therefore, the optimized STS formula could be suggested for local compost production.

## Figures and Tables

**Table 1:** Basic physicochemical properties of experimental soil.

Total N	Total P	Total K	Alkalize N	Available P	Available K	Organic Matter	pH Value	EC Value
g/kg	g/kg	g/kg	mg/kg	mg/kg	mg/kg	g/kg		mS/cm
0.53	0.91	8.60	76.42	117.40	237.70	14.03	8.12	0.242

Abbreviations: Total N: the content of total nitrogen; Total P: the content of total phosphorus; Total K: the content of total potassium; Alkalize N: the content of alkalized nitrogen; Available P: the content of alkalized phosphorus; Available K: the content of available potassium. EC Value: electrical conductivity value.

**Table 2:** Nutrients of raw materials.

Raw Material	Total N	Total P	Total K	Alkalize N	Available P	Available K	Organic Matter
	g/kg	g/kg	g/kg	mg/kg	mg/kg	mg/kg	g/kg
Mushroom residue	9.10	6.65	11.40	630.58	284.33	9253.33	470.31
Cattle manure	9.00	5.71	13.30	686.58	383.29	6786.67	510.76
Sheep manure	8.99	6.61	11.80	658.58	304.87	9320.00	945.67
Corn straw	10.04	7.79	15.09	546.58	385.61	8246.67	998.77
Tail vegetable	11.09	5.41	139.60	971.25	391.25	13,886.67	614.43

Abbreviations: Total N: the content of total nitrogen; Total P: the content of total phosphorus; Total K: the content of total potassium; Alkalize N: the content of alkalized nitrogen; Available P: the content of alkalized phosphorus; Available K: the content of available potassium.

**Table 3:** The basic physicochemical properties of different planting and breeding waste compostings.

Treatments	Total N	Total P	Total K	Alkalize N	Available P	Available K	Organic Matter	pH Value	EC Value	C/N
	g/kg	g/kg	g/kg	mg/kg	mg/kg	mg/kg	g/kg		ms/cm	
ST1	9.19	4.27	14.60	434.58	122.11	7596.67	227.57	8.14	4.81	24.76
ST2	7.03	3.40	15.35	429.92	143.29	8123.33	195.33	7.95	4.92	27.79
STC	9.37	4.12	13.76	415.92	100.87	7013.33	132.75	8.07	5.33	14.17
STM	9.19	4.38	11.68	476.58	93.33	6856.67	176.37	8.03	5.63	19.19
STS	10.13	4.54	14.99	504.58	165.40	8860.00	394.45	8.25	6.35	38.94

Abbreviations: Total N: the content of total nitrogen; Total P: the content of total phosphorus; Total K: the content of total potassium; Alkalize N: the content of alkalized nitrogen; Available P: the content of alkalized phosphorus; Available K: the content of available potassium; EC Value: electrical conductivity value. C/N: carbon-to-nitrogen ratio; ST1 (SM:TV = 6.5:3.5). ST2 (SM:TV = 5.5:4.5). STC (SM:TV:CM = 6:3:1). STM (SM:TV:MR = 6:3:1). STS (SM:TV:CS = 6:3:1).

**Table 4:** Types and performances of used electronic nose sensors.

Numbering	Sensor Type	Response Characteristics
1	W1C	sensitive to aromatic compounds
2	W5S	High sensitivity, sensitive to nitrogen oxides
3	W3C	Sensitive to ammonia and aromatic compounds
4	W6S	sensitive to hydrogen
5	W5C	Sensitive to olefins and aromatic compounds
6	W1S	sensitive to hydrocarbons
7	W1W	sensitive to hydrogen sulfide
8	W2S	Sensitive to alcohols and some aromatic compounds
9	W2W	Sensitive to aromatic compounds and organic sulfur compounds
10	W3S	sensitive to alkanes

**Table 5:** Effects of composting with different formulations of planting and breeding wastes on amino acid composition of mini Chinese cabbage (g/kg DW).

Amino Acid	Treatments				
	ST1	ST2	STC	STM	STS
Threonine	1.52 ± 0.06 b	1.86 ± 0.04 a	1.84 ± 0.04 a	1.38 ± 0.01 c	1.90 ± 0.01 a
Isoleucine	3.80 ± 0.07 c	4.41 ± 0.08 b	3.63 ± 0.07 c	2.26 ± 0.05 d	4.86 ± 0.05 a
Tryptophan	2.08 ± 0.12 b	2.57 ± 0.04 a	2.14 ± 0.04 b	2.40 ± 0.03 a	2.50 ± 0.01 a
Valine	5.38 ± 0.05 b	5.84 ± 0.07 a	5.13 ± 0.06 c	4.69 ± 0.02 d	5.71 ± 0.02 a
Phenylalanine	1.58 ± 0.06 a	1.94 ± 0.04 a	1.35 ± 0.57 a	1.44 ± 0.02 a	2.00 ± 0.01 a
Leucine	3.48 ± 0.07 c	4.05 ± 0.05 b	3.26 ± 0.09 d	2.11 ± 0.05 e	4.40 ± 0.06 a
Methionine	2.62 ± 0.08 b	3.35 ± 0.06 a	3.48 ± 0.05 a	2.46 ± 0.01 b	3.47 ± 0.02 a
Lysine	46.35 ± 0.89 ab	54.03 ± 1.07 a	42.30 ± 6.21 b	39.04 ± 4.40 b	54.23 ± 1.08 a
Arginine	11.81 ± 0.40 b	14.39 ± 0.30 a	11.65 ± 0.11 b	9.12 ± 0.13 c	13.35 ± 0.64 a
Tyrosine	0.30 ± 0.01 b	0.38 ± 0.02 ab	0.31 ± 0.01 b	0.15 ± 0.07 c	0.44 ± 0.01 a
Glycine	1.92 ± 0.04 b	2.21 ± 0.03 a	2.19 ± 0.02 a	1.53 ± 0.04 c	2.18 ± 0.04 a
Glutamate	37.85 ± 1.17 b	44.84 ± 0.81 a	34.80 ± 0.50 c	44.86 ± 0.49 a	31.06 ± 0.23 d
Asparagine	4.54 ± 0.15 c	5.43 ± 0.12 a	5.13 ± 0.11 ab	4.83 ± 0.03 bc	5.23 ± 0.12 a
Proline	3.17 ± 0.06 b	3.50 ± 0.04 a	2.61 ± 0.04 d	2.19 ± 0.04 e	2.98 ± 0.03 c
Cysteine	0.11 ± 0.01 bc	0.20 ± 0.05 a	0.13 ± 0.01 abc	0.16 ± 0.02 ab	0.07 ± 0.01 c
Alanine	16.46 ± 0.30 d	28.60 ± 0.36 a	23.67 ± 0.37 b	15.90 ± 0.15 d	21.23 ± 0.05 c
Serine	3.34 ± 0.08 b	3.46 ± 0.02 b	3.29 ± 0.06 b	2.81 ± 0.08 c	3.84 ± 0.06 a
Glutamine	8.17 ± 0.68 a	6.57 ± 0.22 b	9.08 ± 0.43 a	5.51 ± 0.15 b	9.02 ± 0.13 a
TFAs	154.48 c	187.63 a	156.00 bc	142.82 c	168.43 b
EAAs	64.73 bc	75.48 ab	60.98 c	53.37 c	76.56 a
NEAAs	75.86 c	95.19 a	81.19 b	77.92 bc	76.03 c
MAAs	110.45 b	130.62 a	104.46 b	105.53 b	116.35 b
EAAs/TFAAs%	41.9	40.23	39.09	37.37	45.46
MAAs/TFAAs%	71.5	69.62	66.96	73.89	69.08

Note: Data were means ± SE (n = 3) from three independent replications. Different lowercase letters in the same row of the table indicate significant differences between treatments (*p* < 0.05), according to Duncan’s multiplied test. Abbreviations: ST1 (SM:TV = 6.5:3.5). ST2 (SM:TV = 5.5:4.5). STC (SM:TV:CM = 6:3:1). STM (SM:TV:MR = 6:3:1). STS (SM:TV:CS = 6:3:1). TFAA: total free amino acids; EAAs: essential amino acids; NEAAs: non-essential amino acids; MAAs: the medicinal amino acids.

**Table 6:** Effects of composting with different formulations of planting and breeding wastes on polyphenol in mini Chinese cabbage (ug/g DW).

Polyphenols	Treatments				
	ST1	ST2	STC	STM	STS
Protocatechin acid	105.58 ± 0.90 a	99.76 ± 0.98 b	98.26 ± 0.75 bc	96.18 ± 1.02 c	97.59 ± 0.78 bc
Parahydroxybenzoic acid	108.53 ± 1.16 b	100.95 ± 0.82 c	93.64 ± 1.09 d	99.31 ± 1.15 c	116.42 ± 1.12 a
Chlorogenic acid	108.33 ± 0.73 b	101.97 ± 0.77 c	99.07 ± 0.61 c	94.48 ± 0.61 d	129.11 ± 1.72 a
Gallic acid	100.86 ± 0.83 b	104.21 ± 0.83 a	93.48 ± 0.74 c	93.22 ± 1.00 c	106.49 ± 0.89 a
Coumaric acids	96.15 ± 0.96 a	94.68 ± 0.57 ab	94.77 ± 0.85 ab	92.94 ± 0.87 b	96.54 ± 1.14 a
Ferulic acid	107.74 ± 1.10 b	100.15 ± 1.11 d	95.78 ± 0.58 e	103.61 ± 0.83 c	116.53 ± 1.16 a
Benzoic acid	96.58 ± 0.91 ab	94.26 ± 1.68 bc	91.90 ± 1.01 c	91.97 ± 1.05 c	98.93 ± 1.06 a
Cinnamic acid	103.55 ± 0.99 b	136.72 ± 1.77 a	98.66 ± 0.89 c	101.48 ± 0.84 bc	136.98 ± 2.04 a
Caffeic acid	101.83 ± 0.84 b	96.20 ± 0.66 c	93.78 ± 0.70 cd	93.20 ± 1.09 d	112.72 ± 1.03 a
Sinapic acid	124.01 ± 3.18 c	255.79 ± 2.91 b	120.48 ± 4.91 c	102.90 ± 1.49 d	286.71 ± 4.50 a
Gentisic acid	109.44 ± 0.57 b	96.55 ± 0.79 c	95.22 ± 0.64 c	89.37 ± 0.20 d	143.83 ± 2.27 a
Rutin	283.19 ± 3.23 a	174.14 ± 0.73 b	139.00 ± 1.01 e	148.55 ± 2.59 d	160.97 ± 1.48 c
Quercetin	1154.47 ± 20.43 a	901.10 ± 17.72 b	773.56 ± 19.38 c	855.20 ± 11.63 b	1154.98 ± 7.49 a
Cynarin	95.94 ± 1.01 c	91.42 ± 0.72 c	93.63 ± 0.19 c	91.40 ± 0.54 b	110.82 ± 0.74 a
Kaempferol	95.36 ± 0.48 b	96.10 ± 1.47 b	94.33 ± 0.34 b	94.00 ± 0.91 b	101.48 ± 0.86 a
Total phenolic acids	1138.37 ± 3.05 c	1237.48 ± 1.61 b	1071.94 ± 5.48 d	1054.70 ± 2.92 d	1441.84 ± 11.39 a
Total flavonoids	1628.95 ± 22.16 a	1262.76 ± 19.96 b	1100.52 ± 18.11 d	1189.15 ± 14.66 c	1656.15 ± 9.52 a
Total polyphenols	2767.32 ± 21.03 b	2500.24 ± 21.29 c	2172.46 ± 19.12 e	2243.85 ± 15.75 d	3097.99 ± 14.70 a

Note: Data were means ± SE (n = 3) from three independent replications. Different lowercase letters in the same row of the table indicate significant differences between treatments (*p* < 0.05), according to Duncan’s multiplied test. Abbreviations: ST1 (SM:TV = 6.5:3.5). ST2 (SM:TV = 5.5:4.5). STC (SM:TV:CM = 6:3:1). STM (SM:TV:MR = 6:3:1). STS (SM:TV:CS = 6:3:1).

## Data Availability

The original contributions presented in the study are included in the article. Further inquiries can be directed to the corresponding author.

## References

[1] Li Y., Ma J., Gao X., Tie J., Wu Y., Tang Z., Hu L., Yu J. (2022). Exogenous brassinosteroids alleviate calcium deficiency-induced tip-burn by maintaining cell wall structural stability and higher photosynthesis in mini Chinese Cabbage. Front. Plant Sci..

[2] Sikora J., Niemiec M., Szeląg-Sikora A., Gródek-Szostak Z., Kuboń M., Komorowska M. (2020). The Impact of a Controlled-Release Fertilizer on Greenhouse Gas Emissions and the Efficiency of the Production of Chinese Cabbage. Energies.

[3] Ponjičan O., Kiss F., Ilin Ž., Adamović B., Sabadoš V., Sedlar A., Višacki V. (2021). Influence of plastic mulch and fertilization on the environmental impact of spring cabbage production. Eur. J. Agron..

[4] Sun Q., Ruan Y., Chen P., Wang S., Liu X., Lian B. (2019). Effects of mineral-organic fertilizer on the biomass of green Chinese cabbage and potential carbon sequestration ability in karst areas of Southwest China. Acta Geochim..

[5] Jin L., Jin N., Wang S., Li J., Meng X., Xie Y., Wu Y., Luo S., Lyu J., Yu J. (2022). Changes in the microbial structure of the root soil and the yield of Chinese baby cabbage by chemical fertilizer reduction with bio-organic fertilizer application. Microbiol. Spectr..

[6] Li X., Li B., Chen L., Liang J., Huang R., Tang X., Zhang X., Wang C. (2022). Partial substitution of chemical fertilizer with organic fertilizer over seven years increases yields and restores soil bacterial community diversity in wheat–rice rotation. Eur. J. Agron..

[7] Tian S., Zhu B., Yin R., Wang M., Jiang Y., Zhang C., Li D., Chen X., Kardol P., Liu M. (2022). Organic fertilization promotes crop productivity through changes in soil aggregation. Soil Biol. Biochem..

[8] Qiao Y., Tie J., Wang X., Wei B., Zhang W., Liu Z., Zhang G., Lyu J., Liao W., Hu L. (2023). Comprehensive evaluation on effect of planting and breeding waste composts on the yield, nutrient utilization, and soil environment of baby cabbage. J. Environ. Manag..

[9] Tie J., Qiao Y., Jin N., Gao X., Liu Y., Lyu J., Zhang G., Hu L., Yu J. (2023). Yield and Rhizosphere Soil Environment of Greenhouse Zucchini in Response to Different Planting and Breeding Waste Composts. Microorganisms.

[10] Meena A.L., Karwal M., Dutta D., Mishra R. (2021). Composting: Phases and factors responsible for efficient and improved composting. Agric. Food E-Newsl..

[11] Hidalgo D., Corona F., Martín-Marroquín J.M. (2022). Correction to: Nutrient recycling: From waste to crop. Biomass Convers. Biorefinery.

[12] Baiamonte G., De Pasquale C., Marsala V., Cimò G., Alonzo G., Crescimanno G., Conte P. (2015). Structure alteration of a sandy-clay soil by biochar amendments. J. Soil Sediments.

[13] Kortei N.K., Dzogbefia V.P., Obodai M. (2014). Assessing the Effect of Composting Cassava Peel Based Substrates on the Yield, Nutritional Quality, and Physical Characteristics of *Pleurotus ostreatus* (Jacq. ex Fr.) Kummer. Biotechnol. Res. Int..

[14] Kang S.-M., Shaffique S., Kim L.-R., Kwon E.-H., Kim S.-H., Lee Y.-H., Kalsoom K., Aaqil Khan M., Lee I.-J. (2021). Effects of Organic Fertilizer Mixed with Food Waste Dry Powder on the Growth of Chinese Cabbage Seedlings. Environments.

[15] Lang J., Hu J., Ran W., Xu Y., Shen Q. (2012). Control of cotton *Verticillium* wilt and fungal diversity of rhizosphere soils by bio-organic fertilizer. Biol. Fert. Soils.

[16] Yang X., Liu J., McGrouther K., Huang H., Lu K., Guo X., He L., Lin X., Che L., Ye Z. (2016). Effect of biochar on the extractability of heavy metals (Cd, Cu, Pb, and Zn) and enzyme activity in soil. Environ. Sci. Pollut. Res..

[17] Wang Y., Chen L., Su W., Hao Y., Liu H., Sun G., Chen R., Song S. (2021). Effect of Nitrate Concentration on the Growth, Bolting and Related Gene Expression in Flowering Chinese Cabbage. Agronomy.

[18] Wang J., Zhang J., Li J., Dawuda M.M., Ali B., Wu Y., Yu J., Tang Z., Lyu J., Xiao X. (2021). Exogenous Application of 5-Aminolevulinic Acid Promotes Coloration and Improves the Quality of Tomato Fruit by Regulating Carotenoid Metabolism. Front. Plant Sci..

[19] Hu C., Sun D., Yu J., Chen M., Xue Y., Wang J., Su W., Chen R., Anwar A., Song S. (2024). Transcriptome Analysis of Intermittent Light Induced Early Bolting in Flowering Chinese Cabbage. Plants.

[20] Xie Y., Li J., Jin L., Wei S., Wang S., Jin N., Wang J., Xie J., Feng Z., Zhang G. (2022). Combined Straw and Plastic Film Mulching Can Increase the Yield and Quality of Open Field Loose-Curd Cauliflower. Front. Nutr..

[21] Cataldo D.A., Maroon M., Schrader L.E., Youngs V.L. (1975). Rapid colorimetric determination of nitrate in plant tissue by nitration of salicylic acid. Commun. Soil Sci. Plant Anal..

[22] Jin N., Zhang D., Jin L., Wang S., Yang X., Lei Y., Meng X., Xu Z., Sun J., Lyu J. (2023). Controlling water deficiency as an abiotic stress factor to improve tomato nutritional and flavour quality. Food Chem. X.

[23] Hou Y., Wu G. (2017). Nutritionally nonessential amino acids: A misnomer in nutritional sciences. Adv. Nutr..

[24] Wang L., Xiao L., Zheng S., Pang J., Chen J. (2024). Characterization and assessment of free amino acids in different varieties of sugarcane. Ind. Crop. Prod..

[25] Wang S., Li Y., He L., Yang J., Fernie A.R., Luo J. (2022). Natural variance at the interface of plant primary and specialized metabolism. Curr. Opin. Plant Biol..

[26] Ho T.T.K., Tra V.T., Le T.H., Nguyen N.-K.-Q., Tran C.-S., Nguyen P.-T., Vo T.-D.-H., Thai V.-N., Bui X.-T. (2022). Compost to improve sustainable soil cultivation and crop productivity. Case Stud. Chem. Environ. Eng..

[27] Adekiya A.O., Ejue W.S., Olayanju A., Dunsin O., Aboyeji C.M., Aremu C., Adegbite K., Akinpelu O. (2020). Different organic manure sources and NPK fertilizer on soil chemical properties, growth, yield and quality of okra. Sci. Rep..

[28] Lu X., Yang Y., Hong C., Zhu W., Yao Y., Zhu F., Hong L., Wang W. (2022). Optimization of vegetable waste composting and the exploration of microbial mechanisms related to fungal communities during composting. J. Environ. Manag..

[29] Katakula A.A.N., Handura B., Gawanab W., Itanna F., Mupambwa H.A. (2021). Optimized vermicomposting of a goat manure-vegetable food waste mixture for enhanced nutrient release. Sci. Afr..

[30] Li Y., Achinas S., Zhao J., Geurkink B., Krooneman J., Euverink G.J.W. (2020). Co-digestion of cow and sheep manure: Performance evaluation and relative microbial activity. Renew. Energy.

[31] Said M. (2020). Livestock waste and its role in the composting process: A review. IOP Conference Series: Earth and Environmental Science.

[32] Rana M.S., Hashem M.A., Murshed H.M., Bhuiyan M.K.J., Rahman M.M. (2020). Influence of bulking materials on theorganic matter degradation during composting of cattle manure. J. Agric. Food Environ..

[33] Song C., Ye X., Liu G., Zhang S., Li G., Zhang H., Li F., Sun R., Wang C., Xu D. (2023). Comprehensive Evaluation of Nutritional Qualities of Chinese Cabbage (*Brassica rapa* ssp. *pekinensis*) Varieties Based on Multivariate Statistical Analysis. Horticulturae.

[34] Wang L., Zhang S., Li J., Zhang Y., Zhou D., Li C., He L., Li H., Wang F., Gao J. (2022). Identification of key genes controlling soluble sugar and glucosinolate biosynthesis in Chinese cabbage by integrating metabolome and genome-wide transcriptome analysis. Front. Plant Sci..

[35] Zhan Z., Zhang Y., Geng K., Xue X., Deloire A., Li D., Wang Z. (2023). Effects of Vine Water Status on Malate Metabolism and gamma-Aminobutyric Acid (GABA) Pathway-Related Amino Acids in Marselan (*Vitis vinifera* L.) Grape Berries. Foods.

[36] Rana S., Thakur K.S., Bhardwaj R.K., Kansal S., Sharma R. (2020). Effect of biofertilizers and micronutrients on growth and quality attributes of cabbage (*Brassica oleracea* var. *capitata* L.). Int. J. Chem. Stud..

[37] Belbase P., Bc L. (2020). Effects of different fertilizers on yield and vitamin C content of cauliflower (Brassica oleracea var. botrytis)—A review. Asian J. Agric. Hortic. Res..

[38] Chang H.-Q., Zhu X.-h., Jie W., Guo D.-y., Zhang L.-H., Yao F. (2021). Dynamics of microbial diversity during the composting of agricultural straw. J. Integr. Agric..

[39] Xie Y., Zhou L., Dai J., Chen J., Yang X., Wang X., Wang Z., Feng L. (2022). Effects of the C/N ratio on the microbial community and lignocellulose degradation, during branch waste composting. Bioprocess Biosyst. Eng..

[40] Niu M.F., Liu Z.M., Ma J., Qin M.L., Zhao M.H. (2023). Effects of different conditioners on humification of low C/N compost with vegetable waste. Soils Crops.

[41] Xia Z., Ma Q., Yu W., Wang Y., Zhu M., Zhang X., Gao Y., An S., Li S. (2023). The fate of N released from the fixed NH_4_^+^ pool in response to different straw application doses. Geoderma.

[42] Wang J., Zhang B., Wang J., Zhang G., Yue Z., Hu L., Yu J., Liu Z. (2024). Effects of Different Agricultural Waste Composts on Cabbage Yield and Rhizosphere Environment. Agronomy.

[43] Tie J., Gao X., Liu Y., Chen W., Hu L., Yu J., Li T. (2024). Improving the value of planting and breeding waste compost in agricultural applications: A zucchini cultivation case and circular agricultural models analysis. Chem. Eng. J..

[44] Aboyeji C.M., Adekiya A.O., Dunsin O., Agbaje G.O., Olugbemi O., Okoh H.O., Olofintoye T.A.J. (2019). Growth, yield and vitamin C content of radish (*Raphanus sativus* L.) as affected by green biomass of *Parkia biglobosa* and *Tithonia diversifolia*. Agrofor. Syst..

[45] Citak S., Sonmez S. (2010). Effects of conventional and organic fertilization on spinach (*Spinacea oleracea* L.) growth, yield, vitamin C and nitrate concentration during two successive seasons. Sci. Hortic..

[46] Haghighi M., Barzegar Sadeghabad A., Abolghasemi R. (2022). Effect of exogenous amino acids application on the biochemical, antioxidant, and nutritional value of some leafy cabbage cultivars. Sci. Rep..

[47] Nie J., Fu X., Wang L., Xu J., Gao X. (2023). Impact of Monascus purpureus fermentation on antioxidant activity, free amino acid profiles and flavor properties of kelp (*Saccharina japonica*). Food Chem..

[48] Xia F., Zhao Y., Xing M., Sun Z., Huang Y., Feng J., Shen G. (2022). Discriminant Analysis of the Nutritional Components between Organic Eggs and Conventional Eggs: A ^1^H NMR-Based Metabolomics Study. Molecules.

[49] Yao X., Zhou H., Meng H., Ding J., Shen Y., Cheng H., Zhang X., Li R., Fan S. (2021). Amino acid profile characterization during the co-composting of a livestock manure and maize straw mixture. J. Clean. Prod..

[50] Sun C., Wang D., Shen X., Li C., Liu J., Lan T., Wang W., Xie H., Zhang Y. (2020). Effects of biochar, compost and straw input on root exudation of maize (*Zea mays* L.): From function to morphology. Agric. Ecosyst. Environ..

[51] Yao X., Liu Q., Li D. (2024). Mechanism underlying effects of cellulose-degrading microbial inoculation on amino acid degradation and biosynthesis during composting. Bioresour. Technol..

[52] Li C., Lu Z., Qi R., Zhang Z., Lu Y., Zafar M.H., Yang K., Wang M. (2024). Illumina Sequencing and Metabolomic Analysis Explored the Effects of the Mixed Silage of Rice Straw and Chinese Cabbage Waste on Fecal Microorganisms and Metabolites in Hu Sheep. Fermentation.

[53] Li Y., Li S., Du R., Wang J., Li H., Xie D., Yan J. (2021). Isoleucine enhances plant resistance against Botrytis cinerea via jasmonate signaling pathway. Front. Plant Sci..

[54] Wang J., Liu Z., Dou J., Lv J., Jin N., Jin L., Li Z., Zhang B., Tang Z., Yu J. (2022). A Comparative Study on the Nutrients, Mineral Elements, and Antioxidant Compounds in Different Types of Cruciferous Vegetables. Agronomy.

[55] Wei J., Li S., Su T., Zhao J., Jiang Y., Zubarev Y.A., Bi Y. (2022). Phenolic compositions and antioxidant activities of *Hippophae tibetana* and *H. rhamnoides* ssp. sinensis berries produced in Qinghai-Tibet Plateau. Food Chem. X.

[56] Lopes M., Coimbra M.A., Costa M.d.C., Ramos F. (2023). Food supplement vitamins, minerals, amino-acids, fatty acids, phenolic and alkaloid-based substances: An overview of their interaction with drugs. Crit. Rev. Food Sci. Nutr..

[57] Seong G.U., Hwang I.W., Chung S.K. (2016). Antioxidant capacities and polyphenolics of Chinese cabbage (*Brassica rapa* L. ssp Pekinensis) leaves. Food Chem..

[58] Knutsen H.K., Alexander J., Barregård L., Bignami M., Brüschweiler B., Ceccatelli S., Dinovi M., Edler L., Grasl-Kraupp B., EFSA Panel on Contaminants in the Food Chain (CONTAM) (2016). Erucic acid in feed and food. EFSA J..

[59] Wan Y., Liu J., Deng F., Xie Z., Chen Y., Li J., Li D. (2023). Screening of lignin-degrading fungi and bioaugmentation on the directional humification of garden waste composting. Ind. Crop. Prod..

[60] Siddiqui Y., Munusamy U., Naidu Y., Ahmad K. (2020). Integrated effect of plant growth-promoting compost and NPK fertilizer on nutrient uptake, phenolic content, and antioxidant properties of Orthosiphon stamineus and Cosmos caudatus. Hortic. Environ. Biotechnol..

[61] Zhao B., Wang Y., Li L., Ma L., Deng Y., Xu Z. (2023). Adjusting pH of the Secondary Composting Materials to Further Enhance the Lignocellulose Degradation and Promote the Humification Process. Sustainability.

[62] Yang C., Ye Z., Mao L., Zhang L., Zhang J., Ding W., Han J., Mao K. (2022). Analysis of volatile organic compounds and metabolites of three cultivars of asparagus (*Asparagus officinalis* L.) using E-nose, GC-IMS, and LC-MS/MS. Bioengineered.

[63] Gautier H., Diakou-Verdin V., Bénard C., Reich M., Buret M., Bourgaud F., Poëssel J.L., Caris-Veyrat C., Génard M. (2008). How does tomato quality (sugar, acid, and nutritional quality) vary with ripening stage, temperature, and irradiance?. J. Agric. Food Chem..

[64] Li W., Lu X., Li J. (2022). The effect of organic nutrient solution on flavor in ripe cherry tomato fruit-Transcriptome and metabolomic analyses. Environ. Exp. Bot..

